# Re-understanding and focusing on normoalbuminuric diabetic kidney disease

**DOI:** 10.3389/fendo.2022.1077929

**Published:** 2022-12-02

**Authors:** Na An, Bi-tao Wu, Yu-wei Yang, Zheng-hong Huang, Jia-fu Feng

**Affiliations:** ^1^ National Health Commission Key Laboratory of Nuclear Technology Medical Transformation (MIANYANG CENTRAL HOSPITAL), Mianyang, China; ^2^ Departments of Clinical Laboratory, Mianyang Central Hospital, School of Medicine, University of Electronic Science and Technology of China, Mianyang, China; ^3^ College of Medical Technology, Chengdu University of Traditional Chinese Medicine, Chengdu, China

**Keywords:** diabetic kidney disease, diabetes mellitus, normoalbuminuric diabetic kidney disease, diabetic foot, diabetic foot ulcer

## Abstract

Diabetes mellitus (DM) has grown up to be an important issue of global public health because of its high incidence rate. About 25% of DM patients can develop diabetic foot/ulcers (DF/DFU). Diabetic kidney disease (DKD) is the main cause of end-stage kidney disease (ESKD). DF/DFU and DKD are serious complications of DM. Therefore, early diagnosis and timely prevention and treatment of DF/DFU and DKD are essential for the progress of DM. The clinical diagnosis and staging of DKD are mostly based on the urinary albumin excretion rate (UAER) and EGFR. However, clinically, DKD patients show normoalbuminuric diabetic kidney disease (NADKD) instead of clinical proteinuria. The old NADKD concept is no longer suitable and should be updated accordingly with the redefinition of normal proteinuria by NKF/FDA. Based on the relevant guidelines of DM and CKD and combined with the current situation of clinical research, the review described NADKD from the aspects of epidemiology, pathological mechanism, clinical characteristics, biomarkers, disease diagnosis, and the relationship with DF/DFU to arouse the new understanding of NADKD in the medical profession and pay attention to it.

## 1 Introduction

Diabetic kidney disease (DKD), formerly known as diabetic nephropathy (DN), is the most common microvascular complication in patients with diabetes (diabetes mellitus, DM) and one of the leading causes of death in patients with diabetes. Approximately 50% of end-stage kidney disease (ESKD) originates from DKD (1, 2). About 30 to 50% of DM patients in the world will develop to DKD (2, 3), and DKD patients have a higher risk of survival and mortality compared with simple DM patients (4). Since the concept of DKD was first proposed by the American Kidney Foundation in 2007, it has been widely recognized by the medical academic community, and related research has been deepened. Professional terms have also become more precise from overall planning, and more and more relevant markers have been discovered.

Viberti et al. found that the urinary total protein in patients with type 1 diabetes is within the reference interval, while the excretion of urinary albumin (UAlb) increases. Accordingly, they proposed the concept of microalbuminuria (previously known as albuminuria), that is, the albumin excretion rate (AER) is between 30 and 300 mg/d, which is used as a sensitive indicator of early renal damage in diabetes (5). Therefore, the process of DM complicated with chronic kidney disease (CKD) is a typical disease development with clinical manifestations of in turn increased UAlb excretion, microalbuminuria, macroalbuminuria (with reduced glomerular filtration), and ESKD. However, some DM patients show proteinuria in the normal range but were accompanied by renal insufficiency [estimated global filtration rate (EGFR) < 60 ml/(min/1.73 m^2^)] (6). This phenomenon, first noticed by Lane et al. in 1992 (7), is later called normoalbuminuric diabetic kidney disease (NADKD). Subsequently, more and more references have reported that such cases are common clinically (8–13).

## 2 Redefining NADKD

DKD is a clinical diagnosis, and its etiology is initially thought to be proteinuria in DM patients. The definition is independent of traditional diagnostic markers of CKD, such as histological changes or decreased eGFR, and is initially limited to DM patients with macroalbuminuria. Since then, more sensitive methods for detecting urinary albumin have been developed with the innovation of medical testing technology. Compared with the early detection of macroalbuminuria, proteinuria detected by the new technology is called microalbuminuria, and diabetic patients with microalbuminuria are diagnosed with “early kidney disease. some scholars have also referred to NADKD as diabetic kidney disease without albuminuria (8), non-albuminuric renal disease (9), the non-albuminuric form of diabetes kidney disease (10), normoalbuminuric diabetic nephropathy (11), normoalbuminuric diabetes with renal insufficiency(NADRI) (12), or normoalbuminuric renal insufficiency (13) based on its clinical manifestations or characteristics in the process of clinical research due to the lack of uniform nomenclature.

Some scholars have equated urine proteins with urine albumins, which are called non-proteinuric non-proteinuric diabetic nephropathy (DKD) (14). According to clinical practice, it is inappropriate to call NADKD non-albuminuric or without albuminuria, and normal albuminuric is suitable because even healthy people will have some albumin molecules or fragments in their urine more or less. It is difficult to detect by conventional methods due to the small amount.

Traditionally, urine with UAlb excretion of 30-300 mg/d, or random urine concentration of 20-200 mg/L, or AER of 30-300 mg/d, or urinary albumin-creatinine ratio (UACR) of 30-300 mg/g is called microalbuminuria. The working group of urine albumin testing laboratory in the National Kidney Disease Education Program (NKDE) and the International Federation of Clinical Chemistry (IFCC) recommended the unification of microalbuminuria and macroalbuminuria under term urine albumin, and the abandonment of microalbumin to avoid confusion between microalbumin and small albumin molecule (15) (**Table 1**). The Kidney Disease: Improving Global Outcomes (KDIGO) 2012 guidelines for the management of chronic kidney disease also recommended that term microalbuminuria should no longer be used (16). Although terms microalbuminuria and urine microalbuminuria are still used in some fields, the purpose is to alert patients with diabetes and cardiovascular disease to the possibility of developing chronic kidney disease, not only DKD patients (16).

**Table 1:** Change of terminology.

Current terms			Traditional terms
diabetic kidney disease			diabetic nephropathy
kidney disease			renal disease/nephropathy
albuminuria			normal-, micro-, macro-albuminuria
Type 1 diabetics			Type I diabetics
Type 2 diabetics			Type II diabetics
NHADKD	NADKD		NADKD
	LADKD	IADKD	NADKD
	HADKD		MiDKD
	VHDKD		MaDKD

DKD, diabetic kidney disease; NADKD, normoalbuminuric diabetic kidney disease; NHADKD, non-high albuminuria DKD; LADKD, low albuminuric DKD; HADKD, high albuminuric DKD; VHDKD, very-high albuminuric DKD; MiDKD, microalbuminuric DKD; MaDKD, macroalbuminuric DKD.

The National Kidney Foundation (NKF) and the Food and Drug Administration (FDA) redefined albuminuria in 2009 to strengthen the management of proteinuria in patients with chronic kidney disease (17). i) Those with UACR>300 mg/g are called very high albuminuria (previously called macroalbuminuria); ii) those with UACR between 30 and 300 mg/g are called high albuminuria (previously referred to as microalbuminuria); iii) those between 10 and 29 mg/g are called low albuminuria; iv) only those with UACR<10 mg/g are considered for NADKD (**Table 2**).

**Table 2:** Comparison of new and old concepts of albuminuria diabetic kidney disease.

New definition				
Albuminuria	Normoalbuminuria	Low-albuminuria	High-albuminuria	Very High-albuminuria
Albumin level	<10(URL)mg/d	10(URL)~29mg/d	30~300mg/d	>300mg/d
ACR	<10(URL)mg/g	10(URL)~29mg/g	30~300mg/g	>300mg/g
AER	<10(URL)mg/d	10(URL)~29mg/d	30~300mg/d	>300mg/d
UAlb definition	Normal-	Low-	High-	Very High-
DKD concept	NADKD	LADKD	HADKD	VADKD
			IADKD	
	NHADKD			
**Traditional definition**				
Albuminuria	Normoalbuminuria		Microalbuminuria	Macroalbuminuria
Albumin level	<30mg/d		30~300mg/d	>300mg/d
ACR	<30mg/g		30~300mg/g	>300mg/g
AER	<30mg/d		30~300mg/d	>300mg/d
UAlb definition	Normal-		Micro-	Macro-
DKD concept	NADKD		MiDKD	MaDKD

DKD, diabetic kidney disease; NADKD, normoalbuminuric DKD; NHADKD, non-high albuminuria DKD; LADKD, low albuminuric DKD; HADKD, high albuminuric DKD; VHDKD, very-high albuminuric DKD; MiDKD, micro albuminuric DKD; MaDKD, macro albuminuric DKD; IADKD, Increased albuminuria DKD or DKD with increased albuminuria; UAlb, urine albumin; ACR, albumin-creatinine ratio; AER, albumin excretion rate; URL, upper reference limit.

According to this definition of the NKF/FDA working group, the old name NADKD (old) includes two parts of patients with hypoalbuminemia (UACR:10-29mg/g) and normoalbuminuria (UACR<10 mg/g), and its more accurate definition should be non-high albuminuria DKD (NHDKD) (18). Based on this new definition, patients who used to be called NADKD (old) are not actually “normal albumin” DKD in the true sense.

The work completes the following items to distinguish DKD patients with different levels of albuminuria, e.g., introducing a new concept according to the new definition of NKF/FDA, renaming the old name NADKD (old) to NHADKD, and defining patients with UACR between 10 and 29 mg/g as low albuminuric diabetic kidney disease (LADKD), patients with UACR < 10 mg/g as NADKD (it is a new concept unless otherwise stated in the work), DKD patients with high albuminuria as high albuminuria DKD (HADKD), and DKD patients with very high albuminuria as very-high albuminuria DKD (VADKD). According to the new definition, LADKD, HADKD, and VADKD are collectively referred to as increased albuminuria DKD (or DKD with increased albuminuria, IADKD) for description (**Table 2**).

Many scholars still regard microalbuminuria (ACR between 30 and 300 mg/g) as a biochemical marker for the early diagnosis of DKD (19) and identify NADKD as the early stage of albuminuria DKD because of the different division of concepts. However, some patients with DKD have been found to have normoalbuminuria in clinical practice, accompanied by decreased renal function with reduced eGFR (2). Even if the eGFR decreases to the level of stage 3 CKD, they still show normoalbuminuria. Therefore, NADKD can no longer be simply regarded as the early stage of albuminuria DKD, and it is necessary to recognize and pay attention to NADKD.

## 3 Prevalence of NADKD

The prevalence of NADKD in DKD varies with the method of proteinuria (mostly albumin detection) measurement, regions of the patients, inclusion criteria, and the line of judgment for proteinuria, and the results are in a wide variation (**Table 1**). Patients with NADKD account for only 3.6% of patients with DKD (20), whereas it is as high as 77.9% in Ref (21). The proportion of NADKD in DKD is in the range of 20-70% (22–28). The results of a recent meta-analysis show that the overall prevalence of NADKD in patients with type 2 diabetes and renal insufficiency is 45.6%. That is 24.7% in patients with DKD (albuminuria or renal insufficiency) and 8.4% in all patients with type 2 diabetes, which means that albuminuria does not occur in almost half of patients with DKD and patients with reduced eGFR (29).

The reasons for the high prevalence of NADKD might be related to its pathogenesis (see later); however, the point of the matter is that these reports all use ACR > 30 mg/g or UAE > 30 mg/24 h as a line of judgment to discriminate between NADKD and albuminuric DKD. LADKD is judged as NADKD, and it is not clear whether the proportion of LADKD in it is high or low. Therefore, the roughly reliable prevalence of NADKD can only be clarified by reinvestigation based on the latest definition.

## 4 Causes of NADKD generation

### 4.1 Disease factors: pathogenesis of NADKD

NADKD was thought to be an early stage in the classical process of DM concurrent CKD in the past, which may limit the understanding of NADKD. Still many scholars consider LADKD (10 mg/g or URL ≤ ACR < 30 mg/g) as NADKD (old) even now (**Table 1**). Therefore, LADKD may be the early stage of albuminuric DKD. The prevalence of normal albuminuria gradually decreases with the increased duration of DM and none suffers from normal albuminuria in the 30 DM patients with renal insufficiency lasting more than 21 years (29). The results further confirm that LADKD is the early stage of albuminuric DKD. However, urinary albumin may not be detected in patients with NADKD at the later stages of renal injury (21, 29). Therefore, the occurrence of NADKD may be another form of renal pathological changes caused by DM (30–33), and its pathogenesis needs to be further confirmed and elucidated.

NADKD is a heterogeneous disease with extremely complex pathogenesis, which may involve genetic factors (34, 35), sex hormone abnormalities (36), vascular lesions (more macroangiopathy than microangiopathy) (37), glomerular lesions (38), intrarenal arteriosclerosis (39), mild forms of tubular injury (12), acute kidney injury (40), inflammation (24), metabolic syndrome (41), obesity (42), hypertension (43), hyperlipidemia (44, 45), hyperuricemia (45), and the use of renin-angiotensin-aldosterone system inhibitors (29). Therefore, when these high-risk factors are present in patients with DM, we should pay more attention to whether NADKD occurs or may occur.

### 4.2 Laboratory factors: improper or even false detection

UAlb in some urine samples may not be detected due to the laboratory testing methods, which leads to significantly lower test results. The results of nearly 30mg/g in HADKD patients were detected as low as LADKD or NADKD, leading to improper or even false experimental conclusions.

#### 4.2.1 Physiological variation

Individual biological differences can lead to variations in UAlb excretion rates from 4-103% (15). Therefore, different individuals or the same individual’s urine samples are collected at different times, and the results may be different or even very different, especially among different individuals.

#### 4.2.2 Immunoreactivity of UAlb molecules

Although the two main types of methods currently used in clinical laboratories for detecting UAlb are immunological and chromatographic methods, immunological methods (antigen-antibody reactions) still predominate in clinical laboratories. Immunological methods can only detect UAlb with immunoreactivity. However, when plasma Alb flows through the kidney, only < 1% of intact Alb molecules are filtered under lysosomal enzymes, and > 99% of intact Alb molecules are biochemically modified and degraded into Alb-derived fragments of varying molecular sizes. It results in many UAlb molecules without immunoreactivity. Besides, lysosomes in the renal tubules can cause conformational changes in the Alb antigenic determinants, which causes them to form dimers or multimers and intact Alb molecules to lose their immunoreactivity. Urinary Alb molecules that have lost immunoreactivity cannot be detected by immunological methods, which reduces results (23, 24, 46).

#### 4.2.3 Selection of standards

There are currently three main candidate reference substances for UAlb detection (47): a) ERM-DA470 and ERM-DA470K/IFCC; b) purification of human serum albumin; c) 15N-labeled recombinant human serum albumin (15N-rHSA). However, all three substances are based on human serum Alb rather than human UAlb. The urine matrix is completely different from the serum matrix. Therefore, there is no reference substance based on human UAlb, which leads to systematic errors.

#### 4.2.4 Other

Sample collection and storage containers, preservatives, transportation methods, storage time, as well as subject position, exercise, blood pressure, protein intake, body temperature, mood, and drugs (such as penicillin, nifedipine, acetylsalicylic acid, norepinephrine) can affect the detection of UAlb.

## 5 Diagnosis of NADKD

No institution can formulate clear diagnostic criteria for NADKD for the clinician because of our limited understanding of NADKD and different people having different perspectives.

DKD is usually clinically diagnosed based on the presence of proteinuria and/or decreased eGFR in the absence of signs or symptoms of other major causes of kidney damage (48). Nevertheless, patients with NADKD do not have the UACR. So if we only rely on the eGFR to make a diagnosis of NADKD patients, there will be greater difficulties. The eGFR is a calculation indicator based on serum (plasma) creatinine and/or cystatin C. The level of serum creatinine is easily affected by muscle quality, dietary protein intake, hyperlipidemia, hemolysis, and the method of detection (Jaffe’s method or enzymatic method). An eGFR has an obvious lack of reliability (12). Therefore, the diagnosis of NADKD is challenging.

According to relevant guidelines (16, 17, 48), the diagnosis of NADKD should meet at least the following conditions: i) diagnosis of diabetes according to the latest diagnostic criteria of the World Health Organization (WHO) or the American Diabetes Association (ADA); ii) under the following circumstances more than three months: urinary sediment abnormality, or renal tubular injury resulting in electrolytes and other abnormalities, or abnormal histological examinations, or imaging detected structural abnormalities, or a history of kidney transplantation, or eGFR < 60ml/(min/1.73 m^2^); iii) Have an examination once a month and at least twice in three months if AER<30 mg/24 h or ACR<30 mg/g is diagnosed as NADKD. It also can be diagnosed if AER < 10 mg/24h or ACR < 10 mg/g (or the range of reference values established by the local laboratory) (17, 37).

## 6 Biomarkers

**Figure 1:**
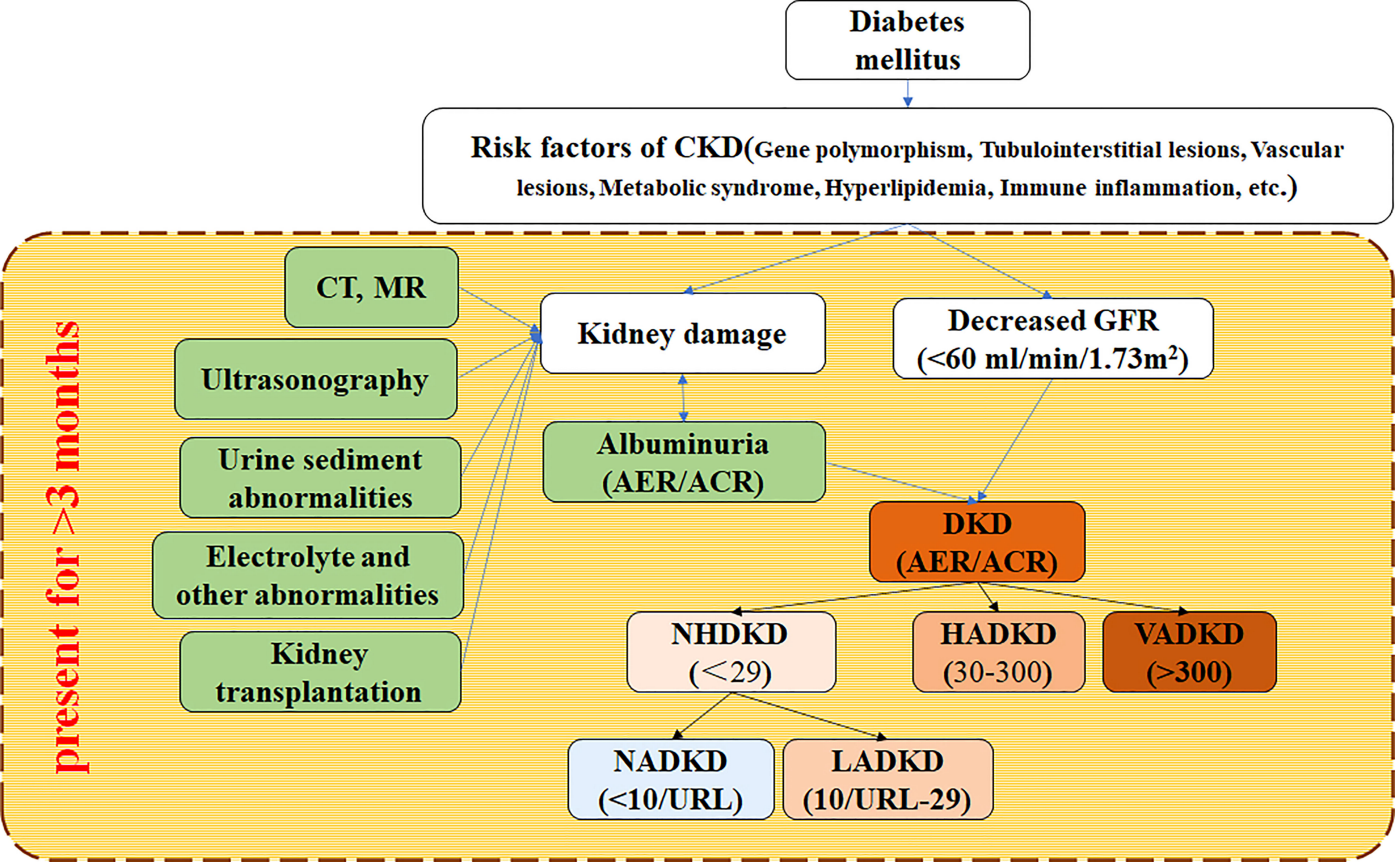
Flow for diagnosing NADKD according to KDIGO 2012 clinical practice guideline KDIGO (2012) defines CKD as abnormalities of kidney structure or function more than 3 months, which affects health. CKD is diagnosed according to markers of kidney injury and decreased GFR. DKD is a kind of CKD. CKD, chronic kidney disease; DKD, diabetic kidney disease; NADKD, normoalbuminuric DKD; NHADKD, non-high albuminuria DKD; LADKD, low albuminuric DKD; HADKD, high albuminuric DKD; VHDKD, very-high albuminuric DKD; CT, computerized tomography; MR, magnetic resonance imaging; ACR, urine albumin-creatinine ratio(unit: mg/g creatinine); AER, albumin excretion rate (unit: mg/24h); URL, upper reference limit.

As far as kidney damage is concerned, the DKD essence is also a chronic kidney disease (CKD). NADKD is only the case part of DKD with normal albuminuria. KDIGO defines CKD as an abnormality of the renal structure and function lasting more than 3 months, which affects human health. According to it, renal structural abnormalities can be found by histological (renal biopsy) or imaging (CT, MR, or ultrasonic) examination. The method cannot determine whether the patient has proteinuria (or albuminuria), so ACR/AER is required to distinguish DKD and NADKD. Therefore, the diagnosis of DKD and NADKD by clinicians mainly depends on the detection of ACR/AER and eGFR in the traditional renal function index (**Figure 1**).

Not all clinical laboratories in other countries reported eGFR except some clinical laboratories in Europe and America because of the tedious detection methods (AER), multiple influencing factors (creatinine) (16), and different degrees of popularity (cystatin C) (16) with no special requirements. The sensitivity of traditional biomarkers (such as ACR/AER and eGFR) for CKD diagnosis is poor, indicating that it is difficult to diagnose DKD/NADKD. Only relying on traditional biomarkers cannot meet the clinical application of NADKD. Therefore, some novel biomarkers emerge.

There are nearly 100 clinical markers for research, screening, or diagnosis of NADKD (**Supplementary Tables 1, 2**). However, only the neutrophil gelatinase-associated lipocalin, cystatin C, vascular endothelial growth factor, and liver-type fatty acid-binding protein can achieve routine testing in clinical laboratories. These projects also have the most research and clinical applications in DKD/NADKD.

### 6.1 Neutrophil gelatinase-associated lipocalin

Neutrophil gelatinase-associated lipocalin (NGAL) is a small molecular protein weighing about 25kDa in the lipid carrier protein superfamily (49). NGAL, produced in ischemic proximal renal tubular cells and participates in the regeneration and repair of proximal renal tubular cells after ischemic renal injury, can reduce the apoptosis of renal tubular cells. Therefore, it can be used as an early biomarker of urine in patients with ischemic renal injury. NGAL in serum and urine may be an early predictive biomarker of acute renal injury (50). The increased level of NGAL in urine indicates that there may be an early renal tubular injury, which occurs earlier than albuminuria (51, 52). The level of urinary NGAL is positively correlated with the concentration of urinary albumin in T2DM patients with albuminuria, showing that urinary NGAL can predict disease progression in T2DM patients and is an important predictor independent of UACR and eGFR (53–57). The level of NGAL for T1DM patients will be increased before the appearance of microalbuminuria, which can evaluate the early renal involvement in the course of diabetes (11). A large number of studies have shown that the NGAL level in blood and urine is significantly higher in patients with NADKD than in healthy controls and/or in patients with DM, suggesting that NGAL in blood and urine is helpful for early diagnosis, disease stage, and prognosis evaluation of NADKD (13, 20, 39, 40, 43, 45, 52, 58–60).

### 6.2 Cystatin C

Cystatin C can be used to calculate eGFR and diagnose renal function damage. According to KIDIGO recommendations, when the eGFR value of patients is 45–59 ml/min/1.73 m^2^ based on creatinine tests, cystatin C should be tested to diagnose or rule out CKD (16). Cystatin C is a cysteine protease inhibitor of 13-kDa, which is produced by nucleated cells at a constant rate and can be filtered freely from the glomerulus. Almost all of it (more than 99%) is reabsorbed and degraded in the proximal convoluted tubule epithelium without returning to the blood circulation as a prototype. So the cystatin C level in the blood mainly depends on eGFR (61, 62). the cystatin C level for DKD patients with albuminuria will increase before the occurrence of albuminuria. Therefore, it is an early diagnostic marker of DKD (63–65). Moreover, a large number of studies demonstrate that increased serum cystatin C in NADKD can be used for the diagnosis and prognosis of NADKD (52, 63, 64). Meanwhile, using cystatin C with/without creatinine to calculate eGFR can improve the sensitivity of eGFR diagnosis (12, 66).

### 6.3 Fatty acid-binding protein

Fatty acid-binding protein (FABP) is a group of low molecular proteins weighing about 14-15 kDa. At least 10 subtypes of FABP have been identified in mammals at present, of which the liver-type fatty acid-binding protein(FABP1, L-FABP), heart-type fatty acid-binding protein(FABP3, H-FABP), adipocyte fatty acid-binding protein(FABP4, A-FABP), and epidermal cell type fatty acid-binding protein(FABP5, E-FABP) are associated with DM vasculopathy (mainly L-FABP) (31). These FABPs are the key inflammatory factors associated with DM and play an important role in DM vasculopathy. The L-FABP level in the urine of patients with NADKD is significantly higher than that in normal controls, and it can reflect the severity of DKD (12, 31). Therefore, L-FABP may help diagnose NADKD. Also, FABP can be used to identify high-risk patients with proteinuria (56).

### 6.4 Vascular endothelial growth factor

Vascular endothelial growth factor (VEGF) is an important regulator of angiogenesis and the most important angiogenic factor associated with renal disease (22). it is expressed in glomerular podocytes, distal renal tubules, and collecting ducts and a small amount in proximal renal tubules in the kidney, which maintains the integrity of the glomerular filtration barrier and podocyte activity (29). Injured podocytes will release VEGF stored in the cytoplasm at the early stage of DKD, which improves the permeability of the glomerular filtration membrane. Since the glomerulus is in a state of high filtration for a long time, the VEGF level is bound to increase.

Patients with T2DM have significantly higher serum VEGF levels than controls (26, 27), and so do elevated urinary VEGF levels in children and adolescents with T1DM who are normoalbuminuric and normotensive (28). The excretion value of VEGF is higher than that of the control group and increases with increased ACR in normoalbuminuric T2DM patients (26, 27, 46). Therefore, VEGF may serve as a biomarker for diagnosing DKD occurrence and disease progression, especially in patients without albumin excretion (67). Specifically inhibiting or blocking the expression of VEGF may become a new target for treating DKD (68).

### 6.5 Other biomarkers

#### 6.5.1 Biomarkers of multiple laboratory studies

Although some markers have not been used clinically, at least three or more laboratory studies have reported that they may be potential markers for diagnosing NDAKA. However, research on these markers has not been continued, and some results have no clinical values at present.

Kidney injury molecule-1(KIM-1): KIM-1 is a transmembrane protein whose expression has high tissue specificity, it is highly expressed in renal tubular epithelial cells damaged by ischemia and nephrotoxicity instead of normal renal tissues. Serum KIM-1 levels in NADKD patients are significantly higher than those in healthy controls (31), and KIM-1 levels increase with the progression of CKD (32), suggesting that KIM-1 can be used for diagnosing NADKD and disease severity.

Netrin-1: Netrin-1 belongs to an angiogenic factor whose expression is restricted to endothelial cells in normal kidneys and is rarely expressed in renal tubular epithelial cells (34). Netrin-1 is overexpressed and excreted in urine after renal injury (20, 69). Urinary netrin-1 levels in patients with NADKDK are significantly higher than those in healthy controls (21, 30–32) and increase with increased urinary albumin levels (21); however, the changes in serum levels are not significant (32). Therefore, urinary netrin-1 can be used as a diagnostic biomarker to predict NADKD and is closely related to the progression of DKD. The excretion of netrin-1 in T2DM patients with DKD is higher than that in T1DM patients (21).

mircoRNA (miRNA): miRNAs are a class of short noncoding RNAs as well as important post-transcriptional regulators of gene expression. Compared with other organs, kidney tissues express some specific miRNAs (70). miRNA 21 and miRNA 124 are elevated in the urine of NADKD patients (40); the miRNA profiles of NADKD patients are different from those of non-DM controls, among which miRNA 145 and miRNA 130 are higher than those of controls. miRNAs contribute to the diagnosis of NADKD (**Supplementary Table 1**); however, these conclusions are mostly based on single-center studies. If they are used in clinical practice, they may need to be verified by large-scale multi-centers. Besides, detection requirements of miRNA are relatively high, which hinders its promotion.

Monocyte chemoattractant protein-1(MCP-1): MCP-1 is a chemokine that can be expressed by a variety of cells in the human body, but normal renal tissue cells only secrete a small amount of MCP-1. A small amount of monocyte infiltration is chemotactic to remove harmful substances and protect kidneys. DM occurrence in patients leads to disorders of various metabolic pathways. MCP-1 mRNA and proteins are highly expressed in various renal tissue cells, which aggregates macrophages and myofibroblasts and results in renal tubular damage. The renal damage degree related to serum and urine MCP-1 levels is positively correlated, indicating that MCP-1 is a marker to distinguish NADKD and IADKD at the early stage (71).

Connective tissue growth factor (CTGF): For DM patients, CTGF can affect the physiological function of renal tissue cells, cellular inflammatory responses, matrix collagen synthesis, and fibronectin synthesis, induce cell apoptosis, and promote fibroblast differentiation, which results in renal injury. Early studies on NADKD show that the serum MCP-1 level is positively correlated with the albumin excretion rate, indicating that it plays an important role in the occurrence and development of DKD (72).

Periostin: Periostin is originally isolated from a mouse osteoblast cell line and named osteoblast-specific factor 2, which can interact with a variety of cytokines and inflammatory mediators to cause inflammation and tissue fibrosis. A recent study (73) shows that serum periostin levels increase in both NADKD and IADKD and are positively correlated with UACR levels, indicating that Periostin may be a marker of early kidney injury, disease progression, and staging in DKD (38).

#### 6.5.2 Biomarkers of single laboratory studies

Although the understanding of NADKD is unclear, clinical research on it is increasing day by day, and dozens of relevant biomarkers have been discovered and recognized (see **Supplementary Table 2**). However, these markers only come from a single-center laboratory and have no response and support from other laboratories. Whether they are really helpful for the diagnosis, treatment, and/or prognosis of DKD/NADKD needs more and more extensive experiments to verify and evaluate.

## 7 Complications of diabetes

### 7.1 Common complications and mechanisms of DM

Nerve damage, microvascular disease, and large vessel damage are caused by the oxidative stress reaction and pro-inflammatory response in various tissues and organs of diabetes patients due to the long-term continuous influence of hyperglycemia, which damages the heart, brain, liver, and kidney, peripheral nerve, eyes, feet, skin, and other organs damaged with complications. The common ones are diabetes nephropathy, eye disease, foot ulcers, heart disease, peripheral neuropathy, cardiomyopathy, autonomic neuropathy, retinopathy, and osteoporosis (74–76). The work only reviews diabetic foot/ulcer (DF/DFU) and diabetic nephropathy.

### 7.2 DF/DFU and DKD

DF/DFU is a common complication of DM caused by microvascular disease, which can seriously reduce the quality of life (QOL) and survival status of DM patients, including shortening the survival life (77). DF is defined as a structural or functional change of the foot, which may manifest as ulceration, osteomyelitis, or gangrene (78). DFU is one of the serious clinical manifestations of DF and an important cause of disability (amputation) in DM patients (78–80). The lifetime prevalence of DFU is high, ranging from 19 to 34%, and the ulcer recurrence rate after wound healing is as high as 40% (81–83).

The purpose of treating DFU is to control the disease and improve the QOL and survival of patients with the transformation of living conditions and medical models (84). The relevant studies have shown that the etiology of DFU is complex and diverse, and chronic inflammatory response and oxidative stress are important factors leading to poor wound healing and the formation of chronic open ulcers in DFU (85). Similarly, the pathogenesis of CKD (DKD/NADKD in DM patients), a common complication of DM, is extremely complex. Inflammatory reactions and oxidative stress are important pathological mechanisms of NADKD (12). However, the relationship between the risk of DF/DFU and the occurrence and severity of CKD is not well understood.

Some previous studies have found that CKD plays an important role in promoting the occurrence and development of DF/DFU. Firstly, CKD may act synergistically with diabetes to cause DF/DFU by increasing peripheral nerve damage and other pathways (86). CKD promotes more severe peripheral vascular disease by causing chronic inflammation and oxidative stress and inducing a prothrombotic state. It increases the risks of chronic inflammation, fluid retention, alterations in the renin-angiotensin system, and ischemic ulcers in DM patients, which ultimately leads to foot ulcers (87, 88).

Secondly, patients with DKD/NADKD have metabolic disorders and/or increased renal filtration proteins, and the body is prone to hypoalbuminemia, which causes poor ulcer healing. Meanwhile, hypoalbuminemia can lead to foot edema, which affects the blood supply of the affected limb and leads to peripheral circulatory disorders. It is more difficult for wound healing in patients with ischemia-based diabetic foot (89).

Thirdly, DKD/NADKD patients may lack trace elements such as zinc, copper, selenium, and nutrients (e.g., vitamins A, C, D, and E and amino acids). The nutritional status is the key factor for general wound healing, the occurrence of DFU, and the prognosis of DFU infection (81, 83, 90, 91).

Fourthly, retinopathy occurs in the process of CKD microangiopathy caused by DM patients, resulting in vision loss. The disturbance of the disease and vision loss will reduce the patient’s mobility and the ability for foot self-care or examination, which increases the possibility of DF and DFU (90).

Both the International Working Group on the Diabetic Foot (IWGDF) and the National Institute for Health and Care Excellence (NICE) agree that patients with DKD are at higher risk for DFU and have a worse prognosis than other patients with diabetes (81, 92). Studies have shown that CKD is related to the severity of DFU patients. DFU patients with CKD have a higher risk of amputation (93). CKD can aggravate the condition of DFU patients and increase the probability of amputation and the risk of death (94, 95). Besides, lower limb complications are the main adverse events of CKD patients, which leads to a higher incidence of DF and a worse prognosis of DFU (96). Compared with CKD patients without DFU, CKD patients with foot ulcers have significantly lower eGFR; compared with patients with pre-existing CKD, the eGFR reduction in DFU patients with no early signs of CKD is also slowly progressive (97).

Since NADKD does not produce proteinuria, its clinical manifestations are more insidious. According to the existing diagnostic methods, it is easy to miss the diagnosis. Therefore, more attention should be paid to NADKD patients, especially some NADKD patients who do not develop proteinuria for life (98). Although patients with DKD are more likely to develop foot ulcers, they are not less likely to heal than diabetic patients with normal renal function (99). Therefore, there are sufficient clinical observations to support the feasibility of salvaging DF/DFU patients even in these high-risk groups.

## 8 Conclusions

DKD is a common serious complication in DM patients, which is associated with renal failure, cardiovascular disease, and premature death. The pathological mechanism of DM complicated with CKD (i.e., DKD occurs) is extremely complex. The proportion of NADKD in DM or DKD patients has gradually increased. Although this may be related to the improvement of the therapeutic effect of DM and the potential renal pathological changes of NADKD, it brings greater difficulties to the diagnosis of DKD. NADKD was judged at the early stage of typical DKD in the past, which might be related to the disease stratification of NADKD. NADKD was falsely judged as NADKD.

DF/DFU is also a serious complication of DM. If the influence of renal injury on DF/DFU disease is ignored due to the missed diagnosis of NADKD, it will certainly aggravate the disease development and lead to serious outcomes for DM patients. Therefore, early diagnosis is essential to improve the prognosis of DM patients. Besides traditional (ACR/AER and eGFR) markers, some novel markers with higher sensitivity and better specificity, such as serum cystatin C, NGAL, L-FABP, and VEGF, should be used as routine evaluation indicators in the clinical management of DM. They can be used to predict the occurrence of DM complications and disease progression through regular examination, screen NADKD patients with concealed clinical manifestations as early as possible, give personalized intervention and/or treatment in time, manage DM, and control the occurrence and development of its complications.

## Author contributions

NA and J-FF contributed to the conception and design of the article. NA, B-TW, and J-FF wrote the first draft of the manuscript. Y-WY and Z-HH performed the framework, tables and the figure. NA and J-FF revised important intellectual content critically for important intellectual content. All authors contributed to the article and approved the submitted version.

## Funding

This work was supported by the Science and Technology Department of Sichuan Province (2015SZ0117, 2019YJ0701, and 21YYJC3525).

## Acknowledgements

We would like to give our sincere appreciation to the reviewers for their helpful comments on this.

## Conflict of interest

The authors declare that the research was conducted in the absence of any commercial or financial relationships that could be construed as a potential conflict of interest.

## Publisher’s note

All claims expressed in this article are solely those of the authors and do not necessarily represent those of their affiliated organizations, or those of the publisher, the editors and the reviewers. Any product that may be evaluated in this article, or claim that may be made by its manufacturer, is not guaranteed or endorsed by the publisher.
