# Supplementary material for: Re-understanding and focusing on normoalbuminuric diabetic kidney disease
Source: Front Endocrinol (Lausanne). 2022 Dec 2;13:1077929. doi: 10.3389/fendo.2022.1077929 (PMC9757068; doi:10.3389/fendo.2022.1077929)
Supplement: Supplementary file 1 [file Table_1.docx]

**Supplementary Table 1 Main research and clinical application of NADKD related biomarkers**

| Biomarker | Sample type | Type of DM(n/female) | eGFR method | AER/ACR cutoff values | First author [Ref.] |
| --- | --- | --- | --- | --- | --- |
| Cys C | serum | T1(29/11) | Cys C-based | ACR<30 mg/g | Salem NA [S1] |
| Cys C | serum | T2(26/16) | MDRD | ACR<30 mg/g | Al-Hazmi SF [S2] |
| CysC | urine | T2(29/–) | CKD-EPI | ACR<30 mg/g | Hassan M [S3] |
| Cys C | serum | T2(29/17) | MDRD, CKD-EPI | ACR<30 mg/g | Jeon YK [S4] |
| Cys C | urine | T2(29/17) | MDRD, CKD-EPI | ACR<30 mg/g | Jeon YK [S4] |
| Cys C | serum | T2(20/10) | MDRD | AER<30 mg/dL | Takir M [S5] |
| Cys C | urine | – | MDRD | ACR<30 mg/g | Borges RL [S6] |
| Cys C | Urine | T2(90/40) | – | ACR<30mg/g | Siddiqi Z [S7] |
| Cys C | Serum | T2(–/–) | MDRD | – | Takir M [S5] |
| Cys C | serum | – | Cockcroft-Gault | – | Asefy Z [S8] |
| Cys C | plasma | T1(32/–) | MDRD | – | Cherney DZ [S9] |
| Cys C | serum | T2(61/–) | – | – | Borges RL [S6] |
| CTGF | plasma | T2(18/11) | Cockcroft-Gault | AER<30 mg/l | El Mesallamy HO [S10] |
| CTGF | urine | T1(30/–) | – | – | Ellina O [S11] |
| CTGF | plasma | T1(188/127) | MDRD | AER<30 mg/d | Nguyen TQ [S12] |
| KIM-1 | urine | T2(58/–) | – | – | Kim SS [S13] |
| KIM-1 | plasma | T1/T2(54/26) | Schwartz | ACR<10 mg/g | Ahn MB [S14] |
| KIM-1 | urine | T1(62/31 | – | – | Abd El Dayem S [S15] |
| KIM-1 | urine | T2(64/43) | – | ACR<10 mg/g | de Carvalho JA [S16] |
| KIM-1 | serum | T2(191/110) | CKD-EPI | ACR<30 mg/g | Colombo M [S17] |
| H-FABP | urine | T1,T2(41/11) | MDRD | ACR<26 mg/g | Nauta FL[S18] |
| L-FABP | urine | T1(29/–) | – | – | Suh JS [S19] |
| L-FABP | urine | T2(466/–) | – | ACR<30 mg/g | Ito H [S20] |
| L-FABP | urine | T2(58/–) | – | – | Kim SS [S13] |
| L-FABP | urine | T1(62/31 | – | – | Abd El Dayem S [S15] |
| miR‑192 | blood | T2(30/18) | MDRD | ACR<30 mg/g | Al-Kafaji G [S21] |
| miR-192 | urine | T2(30/3) | CKD-EPI | ACR<22 mg/g | Jia Y [S22] |
| miR-216a | urine | T1(26/9) | Cockgroft-Gault | ACR<30 mg/g | El-Samahy MH [S23] |
| miR-217 | serum | T2(186/91) | – | ACR<30 mg/g | Shao Y [S24] |
| miR-377 | urine | T1(26/9) | Cockgroft-Gault | ACR<30 mg/g | El-Samahy MH [S23] |
| miR-377 | blood | T2(30/18) | MDRD | ACR<30 mg/g | Al-Kafaji G [S21] |
| MCP-1 | plasma | T2(18/11) | Cockcroft-Gault | AER<30mg/l | El Mesallamy HO [S10] |
| MCP-1 | urine | T2(25/9) | MDRD | ACR<30 mg/g | Shoukry A [S25] |
| MCP-1 | urine | T1(–/–) | – | AER(–) | Fufaa GD [S26] |
| NGAL | urine | T2(44/16) | CKD-EPI | ACR<30 mg/g | Li A [S27] |
| NGAL | urine | T2(26/16) | MDRD | ACR>30 mg/g | Al-Hazmi SF[S2] |
| NGAL | urine | T1(38/–) | Schwartz | ACR<30 mg/g | Hafez MH [S28] |
| NGAL | Serum | T2(90/40) | – | ACR<30mg/g | Siddiqi Z [S7] |
| NGAL | Urine | T2(90/40) | – | ACR<30mg/g | Siddiqi Z [S7] |
| NGAL | serum | T2(56/–) | – | – | Bolignano D [S29] |
| NGAL | urine | T2(56/–) | – | – | Bolignano D [S29] |
| NGAL | plasma | T2(180/–) | – | ACR<30 mg/g | Wu C [S30] |
| NGAL | urine | T2(58/–) | – | – | Kim SS [S13] |
| NGAL | serum | T2(50/–) | – | – | Najafi L [S31] |
| NGAL | urine | T2(50/–) | – | – | Najafi L [S31] |
| NGAL | urine | T1(50/25) | – | – | Lacquaniti A [S32] |
| NGAL | urine | T1(62/31 | – | – | Abd El Dayem S [S15] |
| NGAL | urine | T2(64/43) | – | ACR<10 mg/g | de Carvalho JA [S16] |
| Netrin-1 | urine | T1/T2(40/10) | MDRD | ACR<30 mg/g | Jayakumar C [S33] |
| Netrin-1 | serum | T2(45/24) | MDRD | ACR<30 mg/g | Elkholy RA [S34] |
| Netrin-1 | urine | T2(45/24) | MDRD | ACR<30 mg/g | Elkholy RA [S34] |
| Netrin-1 | plasma | T2(–/–) | – | ACR<30 mg/g | Ay E [S35] |
| Netrin-1 | urine | T1(82/–) | – | – | Uçaktürk SA [S36] |
| Periostin | urine | T2(114/52) | CKD-EPI | ACR<30mg/g | Satirapoj B [S37] |
| Periostin | tissue | T2(114/52) | CKD-EPI | ACR<30mg/g | Satirapoj B [S37] |
| periostin | serum | T2(19/7) | – | ACR<30 mg/g | El-Dawla NMQ [S38] |
| VEGF-A | serum | T2(63/24) | MDRD | ACR<30 mg/g | Kacso IM [S39] |
| VEGF | blood | T2(35/–) | CKDEPI | ACR<30 mg/g | Aly MH [S40] |
| VEGF | plasma | T2(201/102) | – | ACR<30 mg/g | Shao Y [S41] |
| VEGF | urine | T1(75/46) | – | ACR<30 mg/g | Dağdeviren Çakır A [S42] |
| VEGF | blood | T2(–/–) | CKD-EPI | – | Aly MH [S40] |

Notes: –，No shown; Cys C, Cystatin C; KIM-1; miR, microRNA; L-FABP, liver type fatty acid-binding protein; NGAL, neutrophil gelatinase-associated lipocalin; VEGF, vascular endothelial growth factor; AER, albumin excretion rate; ACR, albumin-creatinine ratio

**Supplementary Table 2 NADKD related biomarkers in a single center small sample study**

| **No.** | **Biomarker** | **Sample type** | **Type of DM(n/female)** | **eGFR method** | **AER/ACR cutoff values** | **First author [Ref.]** |
| --- | --- | --- | --- | --- | --- | --- |
| 1 | AcSDKP | urine | T2(21/–) | – | – | Nitta K [S43] |
| 2 | ADMA | plasma | T2(183/–) | – | ACR<30 mg/g | Hanai K [S44] |
| 3 | AGEs | plasma | T1(103/–) | – | – | Beisswenger PJ [S45] |
| 4 | AGT | urine | T1(28/11) | Cys C-based | ACR<30 mg/g | Saito T [S46] |
|  | AGT | urine | T1(75/46) | – | ACR<30 mg/g | Dağdeviren Çakır A [S42] |
| 5 | AGP1 | urine | T2(43/20) | – | ACR<30 mg/g | Jin J [S47] |
| 6 | ALK | plasma | T1(313/–) | CKD-EPI | AER<30 mg/d | Bulum T [S48] |
| 7 | AOPPs | plasma | T2(112/–) | – | – | Liang M [S49] |
| 8 | A1AT | urine | T2(43/20) | – | ACR<30 mg/g | Jin J [S47] |
| 9 | Ang-2 | blood | T2(–/–) | CKD-EPI | – | Aly MH [S50] |
|  | Ang-2 | blood | T2(35/–) | CKDEPI | ACR<30 mg/g | Aly MH [S40] |
| 10 | acyl-carnitines | urine | T1(52/–) | – | AER <30 mg/d | van der Kloet FM [S51] |
| 11 | acyl-glycines | urine | T1(52/–) | – | AER<30 mg/d | van der Kloet FM [S51] |
| 12 | adropin | serum | T2(–/–) | – | ACR<30 mg/g | Li B [S52] |
| 13 | BBOX1 | urine | –(16/9) | MDRD | ACR<30 mg/g | Zhou LT [S53] |
| 14 | bradykinin | plasma | T1(–/–) | – | – | Wheelock KM [S54] |
| 15 | CypA | urine | T1(29/11) | Cys C-based | ACR<30 mg/g | Salem NA [S1] |
| 16 | CD27 | serum | T2(191/110) | CKD-EPI | ACR<30 mg/g | Colombo M [S17] |
| 17 | CEL | plasma | T1(103/–) | – | – | Beisswenger PJ [S45] |
| 18 | CML | plasma | T1(103/–) | – | – | Beisswenger PJ [S45] |
| 19 | Collagen-F | urine | T1/T2(35/–) | – | AER<20 mg/min | Zürbig P [S55] |
| 20 | clusterin | serum | T2(191/110) | CKD-EPI | ACR<30 mg/g | Colombo M [S17] |
| 21 | DBP | Serum | T2(30/17) | – | – | Cho EH [S56] |
| 22 | EGFR | serum | T2(19/8) | – | ACR<30 mg/g | El-Shazly AAA [S57] |
| 23 | E-cadherin | serum | T2(19/7) | – | ACR<30 mg/g | El-Dawla NMQ [S38] |
| 24 | FGF21 | serum | T2(44/23) | CKD-EPI | AER<30 mg/d | Esteghamati A[S58] |
| 25 | FN | plasma | T2(18/11) | Cockcroft-Gault | AER<30 mg/l | El Mesallamy HO [S10] |
| 26 | Gas6 | plasma | T2(130/–) | – | – | Li W [S59] |
| 27 | GAGs | Plasma | T1(30/–) | – | – | Ellina O [S11] |
| 28 | 5-HIAA | plasma | T2(106/–) | MDRD | AER<30 mg/d | Saito J [S60] |
| 29 | HIF-1𝛼 | plasma | T2(201/102) | – | ACR<30 mg/g | Shao Y [S41] |
| 30 | HSP72 | urine | T2(15/11) | – | ACR<30 mg/g | El-Horany HE [S61] |
| 31 | hepcidin | urine | T1(–) | – | AER | Fufaa GD [S26] |
| 32 | haptoglobin | urine | T2(234/–) | – | ACR<30 mg/g | Bhensdadia NM [S62] |
| 33 | IL-6 | urine | T2(125/–) | – | ACR <10mg/g | Sangoi MB [S63] |
| 34 | IL-10 | urine | T2(125/–) | – | ACR <10mg/g | Sangoi MB [S63] |
| 35 | IL-18 | serum | T2（28/18） | CKD-EPI | ACR<30 mg/g | Al-Rubeaan K [S64] |
| 36 | IgLC | urine | T2(20/–) | MDRD | AER<30 mg/g | Hassan SB [S65] |
| 37 | IGF-1 | plasma | T2(201/102) | – | ACR<30 mg/g | Shao Y [S41] |
| 38 | Klotho | serum | T2(63/24) | MDRD | ACR<30 mg/g | Kacso IM [S39] |
|  | Klotho | plasma | T2(180/–) | – | ACR<30 mg/g | Wu C [S30] |
| 39 | leptin | serum | T2(44/0) | Schwartz | ACR<30 mg/g | Yassin MM [S66] |
| 40 | MGHI | plasma | T1(103/–) | – | – | Beisswenger PJ [S45] |
| 41 | MHR | blood | T2(72/40) | CKD-EPI | ACR<30mg/g | Karatas A [S67] |
| 42 | MIOX | serum | T2(30/14) | MDRD | ACR＜30 mg/g | Gao P [S68] |
|  | MIOX | urine | T2(30/14) | MDRD | ACR＜30 mg/g | Gao P [S68] |
| 43 | MK | serum | T1(60/31) | Schwartz | ACR<30 mg/g | Metwalley KA [S69] |
| 44 | MMP | urine | T2(20/12) | MDRD | ACR<22 (M), <30 mg/g(F) | Altemtam N [S70] |
| 45 | MMP-9 | serum | T2(25/10) | MDRD | ACR<30 mg/g | Cakirca G [S71] |
| 46 | TIMP-1 | serum | T2(25/10) | MDRD | ACR<30 mg/g | Cakirca G [S71] |
| 47 | α1-microglobulin | serum | T2(191/110) | CKD-EPI | ACR<30 mg/g | Colombo M [17] |
| 48 | β2-microglobulin | urine | T2(190/–) | MDRD | – | Papale M [S72] |
| 49 | MDA | plasma | T2(43/–) | CKD-EPI  MDRD  Cockcroft-Gault | – | Sauriasari R [S73] |
| 50 | Tryptophan-M | urine | T1(52/–) | – | AER <30 mg/d | van der Kloet FM [S51] |
| 51 | NAG | urine | T2(26/16) | MDRD | ACR<30 mg/g | Al-Hazmi SF [S2] |
| 52 | nephrin | urine | T2(26/15) | MDRD | ACR<30 mg/g | Jim B [S74] |
| 53 | 8-OHdG | plasma | T2(44/16) | CKD-EPI | ACR<30 mg/g | Li A [S27] |
| 54 | 8-iso-PGF2α | urine | T2(43/–) | CKD-EPI  MDRD  Cockcroft-Gault | – | Sauriasari R [S73] |
| 55 | 25(OH)VD3 | plasma | T2(201/102) | – | ACR<30 mg/g | Shao Y [S41] |
| 56 | PDX | urine | T2(56/–) | Cockcroft-Gault | ACR<30 mg/g | Kostovska I [S75] |
| 57 | Pentraxin-3 | serum | T2(–/–) | – | ACR<30 mg/g | Li B [S52] |
| 58 | P-selectin | serum | T2（28/18） | CKD-EPI | ACR<30 mg/g | Al-Rubeaan K [S64] |
| 59 | PSCA | urine | T2(43/20) | – | ACR<30 mg/g | Jin J [S47] |
| 60 | PRO-C6 | serum | – | CKD-EPI | AER<30 mg/d | Pilemann-Lyberg S [S76] |
| 61 | RBP | urine | T2(44/16) | CKD-EPI | ACR<30 mg/g | Li A [S27] |
| 62 | RBP4 | serum | T2(23/13) | MDRD | – | Akbay E [S77] |
| 63 | SH3YL1 | plasma | T2(66/34) | CKDEPI | AER<30 mg/d | Choi GS [S78] |
| 64 | Smad3 | urine | T2(245/103) | MDRD | ACR<30 mg/g | Guo K [S79] |
| 65 | suPAR | plasma | T1(667/–) | – | AER<30 mg/d | Theilade S [S80] |
| 66 | syndecan-1 | serum | T2(191/110) | CKD-EPI | ACR<30 mg/g | Colombo M [S17] |
| 67 | TNF-α | plasma | T2(44/16) | CKD-EPI | ACR<30 mg/g | Li A [27] |
| 68 | TGF-β1 | serum | T2(18/11) | Cockcroft-Gault | AER<30 mg/l | El Mesallamy HO [S10] |
|  | TGF-β1 | urine | T2(20/7) | – | ACR<30 mg/g | Satirapoj B [S81] |
| 69 | TNFR1 | serum | T2(191/110) | CKD-EPI | ACR<30 mg/g | Colombo M [S17] |
| 70 | TNFRs | serum | T2(73/31) | MDRD | ACR=11mg/g | Gohda T [S82] |
| 71 | transferrin | urine | T1(75/46) | – | ACR<30 mg/g | Dağdeviren Çakır A [S42] |
| 72 | ubiquitin | urine | T2(190/–) | MDRD | – | Papale M [S72] |
| 73 | uEGF | urine | T2(642/–) | – | – | Betz BB [S83] |
| 74 | ULK-1 | serum | T2(19/8) | – | ACR<30 mg/g | El-Shazly AAA [S57] |
| 75 | Urotensin-II | Serum | T2(23/13) | – | ACR<30mg/g | Tabur S [S84] |
| 76 | VCAM-1 | serum | T2（28/18） | CKD-EPI | ACR<30 mg/g | Al-Rubeaan K [S64] |
| 77 | VDBP | urine | T2(25/9) | MDRD | ACR<30 mg/g | Shoukry A [S25] |
| 78 | vWf, | plasma | T2(201/102) | – | ACR<30 mg/g | Shao Y [S41] |
| 79 | YKL-40 | urine | T1(29/–) | – | – | Suh JS [S19] |
|  | YKL-40 | plasma | T2(75/–) | – | – | Lee JH [S85] |
| 80 | ZAG | urine | T2(6/0) | – | ACR<30 mg/g | Lim SC [S86] |

Notes: –，No shown; AGT, Angiotensinogen; A1AT, alpha-1-antitrypsin; Ang-2, angiopoietin; AOPPs, advanced oxidized protein products;NAG, N-acetyl-beta-D-glucosaminidase；RBP, retinol-binding protein; TNFRs, tumor necrosis factor (TNF) and its receptors; uCypA, urine Cyclophilin-A; TNFa, tumor necrosis factor a; 8-OHdG, 8-hydroxydeoxyguanosine; VCAM-1, vascular cell adhesion molecule-1; IL, interleukin; MMP, matrix metalloproteinases; FGF21, fibroblast growth factor 21; MCP-1, monocyte chemoattractant protein-1; VDBP, vitamin D-binding protein; MIOX, myo-Inositol Oxygenase; PDX, podocalyxin; 5-HIAA, 5-hydroxyindole acetic acid; TGF-β1, Tumor necrosis factor-β1; CTGF, connective tissue growth factor; FN, fibronectin; MHR, monocyte to high-density lipoprotein cholesterol ratio; H-FABP, heart fatty acid-binding protein; IgLC, immunoglobulin light chain; MK, midkine; TIMP-1, tissue inhibitor of metalloproteinase-1; ALK, alkaline phosphatase; HSP72, heat shock protein 72; GAGs, extracellular matrix-associated glycosaminoglycans; CEL, carboxyethyl lysine; CTGF, connective tissue growth factor; MGHI, methylglyoxal hydroimidazolones; CML, carboxymethyl lysine; MDA, malondialdehyde; AER, albumin excretion rate; ACR, albumin-creatinine ratio

**Supplemental References**

[S1] Salem NA, El Helaly RM, Ali IM, Ebrahim HAA, Alayooti MM, El Domiaty HA, et al. Urine Cyclophilin A and serum Cystatin C as biomarkers for diabetic nephropathy in children with type 1 diabetes. *Pediatr Diabetes*(2020) Aug;21(5):846-55. doi: 10.1111/pedi.13019. Epub(2020) Apr 28

[S2] Al-Hazmi SF, Gad HGM, Alamoudi AA, Eldakhakhny BM, Binmahfooz SK, Alhozali AM. Evaluation of early biomarkers of renal dysfunction in diabetic patients. *Saudi Med J*(2020) Jul;41(7):690-7. doi: 10.15537/smj.2020.7.25168

[S3] Hassan M, Hatata EZ, Al-Arman M, Aboelnaga MM. Urine cystatin C as a biomarker of early renal dysfunction in type 2 diabetic patients. *Diabetes Metab Syndr*(2021) 15(4):102152. doi: 10.1016/j.dsx.2021.05.025

[S4] Jeon YK, Kim MR, Huh JE, Mok JY, Song SH, Kim SS, et al. Cystatin C as an early biomarker of nephropathy in patients with type 2 diabetes. *J Korean Med Sci*(2011) 26(2):258-63. doi: 10.3346/jkms.2011.26.2.258

[S5] Takir M, Unal AD, Kostek O, Bayraktar N, Demirag NG. Cystatin-C and TGF-β levels in patients with diabetic nephropathy. *Nefrologia*(2016) 36(6):653-9. doi: 10.1016/j.nefro.2016.06.011

[S6] Borges RL, Hirota AH, Quinto BM, Ribeiro AB, Zanella MT, Batista MC. Is cystatin C a useful marker in the detection of diabetic kidney disease? *Nephron Clin Pract*(2010) 114(2): c127-34. doi: 10.1159/000254385

[S7] Siddiqi Z, Karoli R, Kaul A, Fatima J, Varshney S, Beg MS. Evaluation of neutrophil gelatinase-associated lipocalin and cystatin C as early markers of diabetic nephropathy. *Ann Afr Med*(2017) 16(3):101-6. doi: 10.4103/aam.aam_12_17

[S8] Asefy Z, Mirinejad M, Amirrasooli H, Tagikhani M. Assessing validity of serum cystatin C for predicting metabolic syndrome. *Pak J Biol Sci*(2014) 17(4):582-5. doi: 10.3923/pjbs.2014.582.585

[S9] Cherney DZ, Sochett EB, Dekker MG, Perkins BA. Ability of cystatin C to detect acute changes in glomerular filtration rate provoked by hyperglycaemia in uncomplicated Type 1 diabetes. *Diabet Med*(2010) 27(12):1358-65. doi: 10.1111/j.1464-5491.2010.03121.x

[S10] El Mesallamy HO, Ahmed HH, Bassyouni AA, Ahmed AS. Clinical significance of inflammatory and fibrogenic cytokines in diabetic nephropathy. *Clin Biochem*(2012) 45(9):646-50. doi: 10.1016/j.clinbiochem.2012.02.021

[S11] Ellina O, Chatzigeorgiou A, Kouyanou S, Lymberi M, Mylona-Karagianni C, Tsouvalas E, et al. Extracellular matrix-associated (GAGs, CTGF), angiogenic (VEGF) and inflammatory factors (MCP-1, CD40, IFN-γ) in type 1 diabetes mellitus nephropathy. *Clin Chem Lab Med*(2012) 50(1):167-74. doi: 10.1515/cclm.2011.881

[S12] Nguyen TQ, Tarnow L, Jorsal A, Oliver N, Roestenberg P, Ito Y, et al. Plasma connective tissue growth factor is an independent predictor of end-stage renal disease and mortality in type 1 diabetic nephropathy. *Diabetes Care*(2008) 31(6):1177-82. doi: 10.2337/dc07-2469

[S13] Kim SS, Song SH, Kim IJ, Kim WJ, Jeon YK, Kim BH, et al. Nonalbuminuric proteinuria as a biomarker for tubular damage in early development of nephropathy with type 2 diabetic patients. *Diabetes Metab Res Rev*(2014) 30(8):736-41. doi: 10.1002/dmrr.2546

[S14] Ahn MB, Cho KS, Kim SK, Kim SH, Cho WK, Jung MH, et al. Poor Glycemic Control Can Increase the Plasma Kidney Injury Molecule-1 Concentration in Normoalbuminuric Children and Adolescents with Diabetes Mellitus. *Children* (Basel)(2021) 8(5):417. doi: 10.3390/children8050417

[S15] Abd El Dayem S, El Bohy Ael M, El Shehaby A. Value of the intrarenal arterial resistivity indices and different renal biomarkers for early identification of diabetic nephropathy in type 1 diabetic patients. *J Pediatr Endocrinol Metab*(2016) 29(3):273-9. doi: 10.1515/jpem-2014-0397

[S16] de Carvalho JA, Tatsch E, Hausen BS, Bollick YS, Moretto MB, Duarte T, et al. Urinary kidney injury molecule-1 and neutrophil gelatinase-associated lipocalin as indicators of tubular damage in normoalbuminuric patients with type 2 diabetes. *Clin Biochem*(2016) 49(3):232-6. doi: 10.1016/j.clinbiochem.2015.10.016

[S17] Colombo M, McGurnaghan SJ, Blackbourn LAK, Dalton RN, Dunger D, Bell S, et al. Comparison of serum and urinary biomarker panels with albumin/creatinine ratio in the prediction of renal function decline in type 1 diabetes. *Diabetologia*(2020) 63(4):788-98. doi: 10.1007/s00125-019-05081-8

[S18] Nauta FL, Boertien WE, Bakker SJ, van Goor H, van Oeveren W, de Jong PE, et al. Glomerular and tubular damage markers are elevated in patients with diabetes. *Diabetes Care*(2011) 34(4):975-81. doi: 10.2337/dc10-1545

[S19] Suh JS, Kim SH, Cho KS, Jung IA, Cho WK, Jeon YJ, et al. Urinary markers in the early stage of nephropathy in patients with childhood-onset type 1 diabetes. *Pediatr Nephrol*(2016) 31(4):623-31. doi: 10.1007/s00467-015-3253-9

[S20] Ito H, Yamashita H, Nakashima M, Takaki A, Yukawa C, Matsumoto S, et al. Current Metabolic Status Affects Urinary Liver-Type Fatty-Acid Binding Protein in Normoalbuminuric Patients With Type 2 Diabetes. J Clin Med Res(2017) 9(4):366-73. doi: 10.14740/jocmr2934w

[S21] Al-Kafaji G, Al-Muhtaresh HA. Expression of microRNA‑377 and microRNA‑192 and their potential as blood‑based biomarkers for early detection of type 2 diabetic nephropathy. *Mol Med Rep*(2018) 18(1):1171-80. doi: 10.3892/mmr.2018.9040

[S22] Jia Y, Guan M, Zheng Z, Zhang Q, Tang C, Xu W, et al. miRNAs in Urine Extracellular Vesicles as Predictors of Early-Stage Diabetic Nephropathy. *J Diabetes Res*(2016) 2016:7932765. doi: 10.1155/2016/7932765

[S23] El-Samahy MH, Adly AA, Elhenawy YI, Ismail EA, Pessar SA, Mowafy ME, et al. Urine miRNA-377 and miRNA-216a as biomarkers of nephropathy and subclinical atherosclerotic risk in pediatric patients with type 1 diabetes. *J Diabetes Complications*(2018) 32(2):185-92. doi: 10.1016/j.jdiacomp.2017.10.014

[S24] Shao Y, Ren H, Lv C, Ma X, Wu C, Wang Q. Changes of serum Mir-217 and the correlation with the severity in type 2 diabetes patients with different stages of diabetic kidney disease. *Endocrine*(2017) 55(1):130-8. doi: 10.1007/s12020-016-1069-4

[S25] Shoukry A, Bdeer Sel-A, El-Sokkary RH. Urine monocyte chemoattractant protein-1 and vitamin D-binding protein as biomarkers for early detection of diabetic nephropathy in type 2 diabetes mellitus. *Mol Cell Biochem*(2015) 408(1-2):25-35. doi: 10.1007/s11010-015-2479-y

[S26] Fufaa GD, Weil EJ, Nelson RG, Hanson RL, Knowler WC, Rovin BH, et al. Urinary monocyte chemoattractant protein-1 and hepcidin and early diabetic nephropathy lesions in type 1 diabetes mellitus. *Nephrol Dial Transplant*(2015) 30(4):599-606. doi: 10.1093/ndt/gfv012

[S27] Li A, Yi B, Liu Y, Wang J, Dai Q, Huang Y, et al. Urine NGAL and RBP Are Biomarkers of Normoalbuminuric Renal Insufficiency in Type 2 Diabetes Mellitus. *J Immunol Res*(2019) 15;2019:5063089. doi: 10.1155/2019/5063089

[S28] Hafez MH, El-Mougy FA, Makar SH, Abd El Shaheed S. Detection of an earlier tubulopathy in diabetic nephropathy among children with normoalbuminuria. *Iran J Kidney Dis*(2015) 9(2):126-31.

[S29] Bolignano D, Lacquaniti A, Coppolino G, Donato V, Fazio MR, Nicocia G, et al. Neutrophil gelatinase-associated lipocalin as an early biomarker of nephropathy in diabetic patients. *Kidney Blood Press Res*(2009) 32(2):91-8. doi: 10.1159/000209379

[S30] Wu C, Wang Q, Lv C, Qin N, Lei S, Yuan Q, et al. The changes of serum sKlotho and NGAL levels and their correlation in type 2 diabetes mellitus patients with different stages of urinary albumin. *Diabetes Res Clin Pract*(2014) 106(2):343-50. doi: 10.1016/j.diabres.2014.08.026

[S31] Najafi L, Keshtkar Rajabi S, Pirsaheb S, Keyvani H, Khajavi A, Shati M, et al. Assessment of Serum and Urine Neurophil Gelatinase- Associated Lipocalin (s-NGAL and u-NGAL) Level as a Predictive Factor of Disease Progression in Diabetic Nephropathy in Type 2 DM. *Iran J Kidney Dis*(2021) 15(4):270-8.

[S32] Lacquaniti A, Donato V, Pintaudi B, Di Vieste G, Chirico V, Buemi A, et al. "Normoalbuminuric" diabetic nephropathy: tubular damage and NGAL. *Acta Diabetol*(2013) 50(6):935-42. doi: 10.1007/s00592-013-0485-7

[S33] Jayakumar C, Nauta FL, Bakker SJ, Bilo H, Gansevoort RT, Johnson MH, et al. Netrin-1, a urine proximal tubular injury marker, is elevated early in the time course of human diabetes. *J Nephrol*(2014) 27(2):151-7. doi: 10.1007/s40620-014-0055-2

[S34] Elkholy RA, Younis RL, Allam AA, Hagag RY, Abdel Ghafar MT. Diagnostic efficacy of serum and urinary netrin-1 in the early detection of diabetic nephropathy. *J Investig Med*(2021) 69(6):1189-95. doi: 10.1136/jim-2021-001785

[S35] Ay E, Marakoğlu K, Kizmaz M, Ünlü A. Evaluation of Netrin-1 Levels and Albuminuria in Patients With Diabetes. *J Clin Lab Anal*(2016) 30(6):972-7. doi: 10.1002/jcla.21965

[S36] Uçaktürk SA, Mengen E, Elmaoğulları S, Yücel Ç, A Yılmaz A, Çifci A. Evaluation of the relationship between short-term glycemic control and netrin-1, a urinary proximal tubular injury marker in children with type 1 diabetes. *J Pediatr Endocrinol Metab*(2019) 32(8):851-6

[S37] Satirapoj B, Tassanasorn S, Charoenpitakchai M, Supasyndh O. Periostin as a tissue and urine biomarker of renal injury in type 2 diabetes mellitus. *PLoS One*(2015) 10(4):e0124055. doi: 10.1371/journal.pone.0124055

[S38] El-Dawla NMQ, Sallam AM, El-Hefnawy MH, El-Mesallamy HO. E-cadherin and periostin in early detection and progression of diabetic nephropathy: epithelial-to-mesenchymal transition. *Clin Exp Nephrol*(2019) 23(8):1050-7. doi: 10.1007/s10157-019-01744-3

[S39] Kacso IM, Bondor CI, Kacso G. Soluble serum Klotho in diabetic nephropathy: relationship to VEGF-A. *Clin Biochem*(2012) 45(16-17):1415-20. doi: 10.1016/j.clinbiochem.2012.07.098

[S40] Aly MH, Arafat MA, Hussein OA, Elsaid HH, Abdel-Hammed AR. Study of Angiopoietin-2 and vascular endothelial growth factor as markers of diabetic nephropathy onset in Egyptians diabetic patients with non-albuminuric state. *Diabetes Metab Syndr*(2019) 13(2):1623-7. doi: 10.1016/j.dsx.2019.03.016

[S41] Shao Y, Lv C, Yuan Q, Wang Q. Levels of Serum 25(OH)VD3, HIF-1α, VEGF, vWf, and IGF-1 and Their Correlation in Type 2 Diabetes Patients with Different Urine Albumin Creatinine Ratio. *J Diabetes Res*(2016) 2016:1925424. doi: 10.1155/2016/1925424

[S42]] Dağdeviren Çakır A, Saygılı SK, Canpolat N, Konukoğlu D, Turan H, Çalışkan S, et al. Elevated Urinary VEGF-A, Transferrin, and Angiotensinogen Levels in Normoalbuminuric Children and Adolescents with Type 1 Diabetes: Can They Be Early Markers of Diabetic Kidney Disease? *Horm Res Paediatr*(2021) 94(11-12):426-32. doi: 10.1159/000521447

[S43] Nitta K, Nagai T, Mizunuma Y, Kitada M, Nakagawa A, Sakurai M, et al. N-Acetyl-seryl-aspartyl-lysyl-proline is a potential biomarker of renal function in normoalbuminuric diabetic patients with eGFR ≥ 30 ml/min/1.73 m2. *Clin Exp Nephrol*(2019) 23(8):1004-12. doi: 10.1007/s10157-019-01733-6

[S44] Hanai K, Babazono T, Nyumura I, Toya K, Tanaka N, Tanaka M, et al. Asymmetric dimethylarginine is closely associated with the development and progression of nephropathy in patients with type 2 diabetes. *Nephrol Dial Transplant*(2009) 24(6):1884-8. doi: 10.1093/ndt/gfn716

[S45] Beisswenger PJ, Howell SK, Russell GB, Miller ME, Rich SS, Mauer M. Early progression of diabetic nephropathy correlates with methylglyoxal-derived advanced glycation end products. *Diabetes Care*(2013) 36(10):3234-9. doi: 10.2337/dc12-2689

[S46] Saito T, Urushihara M, Kotani Y, Kagami S, Kobori H. Increased urine angiotensinogen is precedent to increased urine albumin in patients with type 1 diabetes. *Am J Med Sci*(2009) 338(6):478-80. doi: 10.1097/MAJ.0b013e3181b90c25

[S47] Jin J, Ku YH, Kim Y, Kim Y, Kim K, Lee JY, et al. Differential proteome profiling using iTRAQ in microalbuminuric and normoalbuminuric type 2 diabetic patients. *Exp Diabetes Res*(2012) 2012:168602. doi: 10.1155/2012/168602

[S48] Bulum T, Kolarić B, Duvnjak M, Duvnjak L. Alkaline phosphatase is independently associated with renal function in normoalbuminuric type 1 diabetic patients. *Ren Fail*(2014) 36(3):372-7. doi: 10.3109/0886022X.2013.872569

[S49] Liang M, Wang J, Xie C, Yang Y, Tian JW, Xue YM, et al. Increased plasma advanced oxidation protein products is an early marker of endothelial dysfunction in type 2 diabetes patients without albuminuria 2. *J Diabetes*(2014) 6(5):417-26. doi: 10.1111/1753-0407.12134

[S50] Aly MH, Arafat MA, Hussein OA, Elsaid HH, Abdel-Hammed AR. Study of Angiopoietin-2 and vascular endothelial growth factor as markers of diabetic nephropathy onset in Egyptians diabetic patients with non-albuminuric state. *Diabetes Metab Syndr*(2019) 13(2):1623-7. doi: 10.1016/j.dsx.2019.03.016

[S51] van der Kloet FM, Tempels FW, Ismail N, van der Heijden R, Kasper PT, Rojas-Cherto M, et al. Discovery of early-stage biomarkers for diabetic kidney disease using ms-based metabolomics (FinnDiane study). *Metabolomics*(2012) 8(1):109-19. doi: 10.1007/s11306-011-0291-6

[S52] Li B, Tian X, Guo S, Zhang M, Li J, Zhai N, et al. Pentraxin-3 and adropin as inflammatory markers of early renal damage in type 2 diabetes patients. *Int Urol Nephrol*(2020) 52(11):2145-52. doi: 10.1007/s11255-020-02568-x

[S53] Zhou LT, Lv LL, Qiu S, Yin Q, Li ZL, Tang TT, et al. Bioinformatics-based discovery of the urinary BBOX1 mRNA as a potential biomarker of diabetic kidney disease. *J Transl Med*(2019) 28;17(1):59. doi: 10.1186/s12967-019-1818-2

[S54] Wheelock KM, Cai J, Looker HC, Merchant ML, Nelson RG, Fufaa GD, et al. Plasma bradykinin and early diabetic nephropathy lesions in type 1 diabetes mellitus. *PLoS One*(2017) 12(7):e0180964. doi: 10.1371/journal.pone.0180964

[S55] Zürbig P, Jerums G, Hovind P, Macisaac RJ, Mischak H, Nielsen SE, et al. Urinary proteomics for early diagnosis in diabetic nephropathy. *Diabetes*(2012) 61(12):3304-13. doi: 10.2337/db12-0348

[S56] Cho EH, Kim MR, Kim HJ, Lee DY, Kim PK, Choi KM, et al. The discovery of biomarkers for type 2 diabetic nephropathy by serum proteome analysis. *Proteomics Clin Appl*(2007) 1(4):352-61. doi: 10.1002/prca.200600608

[S57] El-Shazly AAA, Sallam AM, El-Hefnawy MH, El-Mesallamy HO. Epidermal growth factor receptor and podocin predict nephropathy progression in type 2 diabetic patients through interaction with the autophagy influencer ULK-1. *J Diabetes Complications*(2019) 33(2):128-33. doi: 10.1016/j.jdiacomp.2018.11.007

[S58] Esteghamati A, Khandan A, Momeni A, Behdadnia A, Ghajar A, Nikdad MS, et al. Circulating levels of fibroblast growth factor 21 in early-stage diabetic kidney disease. *Ir J Med Sci*(2017) 186(3):785-94. doi: 10.1007/s11845-017-1554-7

[S59] Li W, Wang J, Ge L, Shan J, Zhang C, Liu J. Growth arrest-specific protein 6 (Gas6) as a noninvasive biomarker for early detection of diabetic nephropathy. *Clin Exp Hypertens*(2017) 39(4):382-7. doi: 10.1080/10641963.2017.1288739

[S60] Saito J, Suzuki E, Tajima Y, Takami K, Horikawa Y, Takeda J. Increased plasma serotonin metabolite 5-hydroxyindole acetic acid concentrations are associated with impaired systolic and late diastolic forward flows during cardiac cycle and elevated resistive index at popliteal artery and renal insufficiency in type 2 diabetic patients with microalbuminuria. *Endocr J*(2016) 63(1):69-76. doi: 10.1507/endocrj.EJ15-0343

[S61] El-Horany HE, Abd-Ellatif RN, Watany M, Hafez YM, Okda HI. NLRP3 expression and urinary HSP72 in relation to biomarkers of inflammation and oxidative stress in diabetic nephropathy patients. *IUBMB Life*(2017) 69(8):623-30. doi: 10.1002/iub.1645

[S62] Bhensdadia NM, Hunt KJ, Lopes-Virella MF, Michael Tucker J, Mataria MR, Alge JL, et al. Urine haptoglobin levels predict early renal functional decline in patients with type 2 diabetes. *Kidney Int*(2013) 83(6):1136-43. doi: 10.1038/ki.2013.57

[S63] Sangoi MB, de Carvalho JA, Tatsch E, Hausen BS, Bollick YS, Londero SW, et al. Urinary inflammatory cytokines as indicators of kidney damage in type 2 diabetic patients. *Clin Chim Acta*(2016) Sep 1;460:178-83. doi: 10.1016/j.cca.2016.06.028

[S64] Al-Rubeaan K, Nawaz SS, Youssef AM, Al Ghonaim M, Siddiqui K. IL-18, VCAM-1 and P-selectin as early biomarkers in normoalbuminuric Type 2 diabetes patients. *Biomark Med*(2019) 13(6):467-78. doi: 10.2217/bmm-2018-0359

[S65] Hassan SB, Hanna MO. Urine κ and λ immunoglobulin light chains in normoalbuminuric type 2 diabetes mellitus patients. *J Clin Lab Anal*(2011) 25(4):229-32. doi: 10.1002/jcla.20463

[S66] Yassin MM, AbuMustafa AM, Yassin MM. Serum leptin in diabetic nephropathy male patients from Gaza Strip. *Diabetes Metab Syndr*(2019) 13(2):1245-50. doi: 10.1016/j.dsx.2019.02.004

[S67] Karatas A, Turkmen E, Erdem E, Dugeroglu H, Kaya Y. Monocyte to high-density lipoprotein cholesterol ratio in patients with diabetes mellitus and diabetic nephropathy. *Biomark Med*(2018) 12(9):953-9. doi: 10.2217/bmm-2018-0048

[S68] Gao P, Xu B, Song P, Zhu X, Yuan S, Kanwar YS, Sun L. The Kidney Specific Protein myo-Inositol Oxygenase, a Potential Biomarker for Diabetic Nephropathy. *Kidney Blood Press Res*(2018) 43(6):1772-85. doi: 10.1159/000495635

[S69] Metwalley KA, Farghaly HS, Gabri MF, Abdel-Aziz SM, Ismail AM, Raafat DM, et al. Midkine: Utility as a Predictor of Early Diabetic Nephropathy in Children with Type 1 Diabetes Mellitus. *J Clin Res Pediatr Endocrinol*(2021) 13(3):293-9. doi: 10.4274/jcrpe.galenos.2021.2020.0303

[S70] Altemtam N, Nahas ME, Johnson T. Urine matrix metalloproteinase activity in diabetic kidney disease: a potential marker of disease progression. *Nephron Extra*(2012) (1):219-32. doi: 10.1159/000339645

[S71] Cakirca G, Turgut FH. Serum Matrix Metalloproteinase-9, Tissue Inhibitor of Metalloproteinase-1 and Matrix Metalloproteinase-9/ Neutrophil Gelatinase-associated Lipocalin Complex Levels in Patients With Early-stage Diabetic Nephropathy. *Iran J Kidney Dis*(2018) 12(5):299-304.

[S72] Papale M, Di Paolo S, Magistroni R, Lamacchia O, Di Palma AM, De Mattia A, et al. Urine proteome analysis may allow noninvasive differential diagnosis of diabetic nephropathy. *Diabetes Care*(2010) 33(11):2409-15. doi: 10.2337/dc10-0345

[S73] Sauriasari R, Andrajati R, Azizahwati, Dharmeizar, Saputri DA, Muris RU, et al. Marker of lipid peroxidation related to diabetic nephropathy in Indonesian type 2 diabetes mellitus patients. *Diabetes Res Clin Pract*(2015) 108(1):193-200. doi: 10.1016/j.diabres.2014.12.016

[S74] Jim B, Ghanta M, Qipo A, Fan Y, Chuang PY, Cohen HW, et al. Dysregulated nephrin in diabetic nephropathy of type 2 diabetes: a cross sectional study. *PLoS One*(2012) 7(5):e36041. doi: 10.1371/journal.pone.0036041

[S75] Kostovska I, Trajkovska KT, Cekovska S, Topuzovska S, Kavrakova JB, Spasovski G, et al. Role of urine podocalyxin in early diagnosis of diabetic nephropathy. *Rom J Intern Med*(2020) 58(4):233-41. doi: 10.2478/rjim-2020-0023

[S76] Pilemann-Lyberg S, Rasmussen DGK, Hansen TW, Tofte N, Winther SA, Holm Nielsen S et al. Markers of Collagen Formation and Degradation Reflect Renal Function and Predict Adverse Outcomes in Patients With Type 1 Diabetes. *Diabetes Care*(2019) 42(9):1760-8. doi: 10.2337/dc18-2599

[S77] Akbay E, Muslu N, Nayir E, Ozhan O, Kiykim A. Serum retinol binding protein 4 level is related with renal functions in Type 2 diabetes. *J Endocrinol Invest*(2010) 33(10):725-9. doi: 10.1007/BF03346678

[S78] Choi GS, Min HS, Cha JJ, Lee JE, Ghee JY, Yoo JA, et al. SH3YL1 protein as a novel biomarker for diabetic nephropathy in type 2 diabetes mellitus. *Nutr Metab Cardiovasc Dis*(2021) 31(2):498-505. doi: 10.1016/j.numecd.2020.09.024

[S79] Guo K, Lu J, Kou J, Wu M, Zhang L, Yu H, et al. Increased urine Smad3 is significantly correlated with glomerular hyperfiltration and a reduced glomerular filtration rate and is a new urine biomarker for diabetic nephropathy. *BMC Nephrol*(2015) 16:159. doi: 10.1186/s12882-015-0156-8

[S80] Theilade S, Lyngbaek S, Hansen TW, Eugen-Olsen J, Fenger M, Rossing P, et al. Soluble urokinase plasminogen activator receptor levels are elevated and associated with complications in patients with type 1 diabetes. *J Intern Med*(2015) 277(3):362-71. doi: 10.1111/joim.12269

[S81] Satirapoj B, Kaewput W, Supasyndh O, Ruangkanchanasetr P. Effect of sulodexide on urinary biomarkers of kidney injury in normoalbuminuric type 2 diabetes: a randomized controlled trial. *J Diabetes Res*(2015) 2015:172038. doi: 10.1155/2015/172038

[S82] Gohda T, Nishizaki Y, Murakoshi M, Nojiri S, Yanagisawa N, Shibata T, et al. Clinical predictive biomarkers for normoalbuminuric diabetic kidney disease. *Diabetes Res Clin Pract*(2018) 141:62-8. doi: 10.1016/j.diabres.2018.04.026

[S83] Betz BB, Jenks SJ, Cronshaw AD, Lamont DJ, Cairns C, Manning JR, et al. Urinary peptidomics in a rodent model of diabetic nephropathy highlights epidermal growth factor as a biomarker for renal deterioration in patients with type 2 diabetes. *Kidney Int*(2016) 89(5):1125-35. doi: 10.1016/j.kint.2016.01.015

[S84] Tabur S, Korkmaz H, Eren MA, Oğuz E, Sabuncu T, Aksoy N. Urotensin-II level and its association with oxidative stress in early diabetic nephropathy. *J Diabetes Complications*(2015) 29(1):115-9. doi: 10.1016/j.jdiacomp.2014.07.011

[S85] Lee JH, Kim SS, Kim IJ, Song SH, Kim YK, In Kim J et al. Clinical implication of plasma and urine YKL-40, as a proinflammatory biomarker, on early stage of nephropathy in type 2 diabetic patients. *J Diabetes Complications*(2012) 26(4):308-12. doi: 10.1016/j.jdiacomp.2012.04.012

[S86] Lim SC, Liying DQ, Toy WC, Wong M, Yeoh LY, Tan C, et al. Adipocytokine zinc α2 glycoprotein (ZAG) as a novel urinary biomarker for normo-albuminuric diabetic nephropathy. *Diabet Med*(2012) 29(7):945-9. doi: 10.1111/j.1464-5491.201
